# Supplementary material for: Cryo-EM reveals how Hsp90 and FKBP immunophilins co-regulate the glucocorticoid receptor
Source: Nat Struct Mol Biol. 2023 Nov 9;30(12):1867–77. doi: 10.1038/s41594-023-01128-y (PMC10716051; doi:10.1038/s41594-023-01128-y)
Supplement: Supplementary file 1 — Reporting Summary [file 41594_2023_1128_MOESM1_ESM.pdf]

nature portfolio

Corresponding author(s): David A. Agard

Last updated by author(s): 09/04/2023

Reporting Summary

Nature Portfolio wishes to improve the reproducibility of the work that we publish. This form provides structure for consistency and transparency in reporting. For further information on Nature Portfolio policies, see our Editorial Policies and the Editorial Policy Checklist.

Statistics

For all statistical analyses, confirm that the following items are present in the figure legend, table legend, main text, or Methods section.

n/a	Confirmed	
☐	☒	The exact sample size (*n*) for each experimental group/condition, given as a discrete number and unit of measurement
☐	☒	A statement on whether measurements were taken from distinct samples or whether the same sample was measured repeatedly
☐	☒	The statistical test(s) used AND whether they are one- or two-sided<br>*Only common tests should be described solely by name; describe more complex techniques in the Methods section.*
☒	☐	A description of all covariates tested
☐	☒	A description of any assumptions or corrections, such as tests of normality and adjustment for multiple comparisons
☐	☒	A full description of the statistical parameters including central tendency (e.g. means) or other basic estimates (e.g. regression coefficient) AND variation (e.g. standard deviation) or associated estimates of uncertainty (e.g. confidence intervals)
☐	☒	For null hypothesis testing, the test statistic (e.g. *F*, *t*, *r*) with confidence intervals, effect sizes, degrees of freedom and *P* value noted<br>*Give P values as exact values whenever suitable.*
☒	☐	For Bayesian analysis, information on the choice of priors and Markov chain Monte Carlo settings
☒	☐	For hierarchical and complex designs, identification of the appropriate level for tests and full reporting of outcomes
☒	☐	Estimates of effect sizes (e.g. Cohen's *d*, Pearson's *r*), indicating how they were calculated

Our web collection on statistics for biologists contains articles on many of the points above.

Software and code

Policy information about availability of computer code

Data collection	Cryo-EM data collection was done on a Titan Krios (Thermo Fischer Scientific) electron microscope (300kV) with a Gatan K3 direct electron detector (Gatan) equipped with a Bioquantum energy filter (Gatan) set to a slit width of 20 eV. Automatic data collection was done with SerialEM v.4.0.
Data analysis	EM data processing was done with RELION v.3.0.8, CryoSparc v.3.3.2, UCSF MotionCor2, and CTFFIND v.4.1. Figures were created with UCSF Chimera v.1.14, UCSF ChimeraX v.1.0.0, and BioRender.com. Model building was done using Rosetta v.3.11 and AlphaFold (https://alphafold.ebi.ac.uk/). Graphical data was plotted and statistical analysis was performed using Prism (GraphPad) v.9.4.0. Sequence alignments were performed using Uniprot, Clutal Omega (https://www.ebi.ac.uk/Tools/msa/clustalo/), and JalView (v.2.11.1.0).

For manuscripts utilizing custom algorithms or software that are central to the research but not yet described in published literature, software must be made available to editors and reviewers. We strongly encourage code deposition in a community repository (e.g. GitHub). See the Nature Portfolio guidelines for submitting code & software for further information.

Data

Policy information about availability of data

All manuscripts must include a data availability statement. This statement should provide the following information, where applicable:

- Accession codes, unique identifiers, or web links for publicly available datasets
- A description of any restrictions on data availability
- For clinical datasets or third party data, please ensure that the statement adheres to our policy

The cryo-EM maps generated in this study have been deposited in the Electron Microscopy Data Bank (EMDB) under the accession codes EMD-29068 (GR:Hsp90:FBKP52) and EMD-29069 (GR:Hsp90:FKBP51). The atomic coordinates have been deposited in the PDB under the accession code 8FFV (GR:Hsp90:FKBP52) and 8FFW (GR:Hsp90:FKBP51). Publicly available PDB entries used in this study are: 7KRJ, 5NJX, 7L7I, 1M2Z, 4LAV, 6TXX and AlphaFold AF-Q02790. Protein sequence data for sequence alignments are available from Uniprot. Sequences in the FKBP51/52 alignment are: H. sapiens FKBP52, M. musculus FKBP52, R. norvegicus FKBP52, D. melanogaster FKBP52, T. guttata FKBP52, G. gallus FKBP52, X. tropicalis FKBP52, and H. sapiens FKBP51 (Uniprot accession codes: Q02790, P30416, Q9QVC8, Q6IQ94, H0ZSE5, A0A3Q3B0L8, A0A310SUH5, Q13451 respectively). Sequences in the GR alignment are: H. sapiens GR, M. musculus GR, R. norvegicus GR, T. guttata GR, G. gallus GR, X. tropicalis GR, D. rerio GR (Uniprot accession codes: P04150, P06537, P06536, A0A674H6U9, A0A1D5PRD7, Q28E31, A0A2R8QN75, respectively). Sequences in the human steroid hormone receptor alignment are: glucocorticoid receptor, mineralocorticoid receptor, androgen receptor, progesterone receptor, estrogen receptor α and β (Uniprot accession codes: P04150, P08235, P10275, P06401, E3WH19, Q92731, respectively).

Human research participants

Policy information about studies involving human research participants and Sex and Gender in Research.

Reporting on sex and gender	N/A
Population characteristics	N/A
Recruitment	N/A
Ethics oversight	N/A

Note that full information on the approval of the study protocol must also be provided in the manuscript.

Field-specific reporting

Please select the one below that is the best fit for your research. If you are not sure, read the appropriate sections before making your selection.

☒ Life sciences ☐ Behavioural & social sciences ☐ Ecological, evolutionary & environmental sciences

For a reference copy of the document with all sections, see nature.com/documents/nr-reporting-summary-flat.pdf

Life sciences study design

All studies must disclose on these points even when the disclosure is negative.

Sample size	No statistical method was used for sample size calculation. All in vitro biochemical experiments were performed using three biological replicates to account for pipetting variability. All in vivo yeast assays were performed using three biological replicates to account for pipetting variability and biological variability between yeast cultures. For cryoEM reconstructions, sample sizes were determined by available electron microscopy time and the number of particles on each micrograph obtained during the collection time.
Data exclusions	No data was excluded.
Replication	All experiments were confirmed with multiple biological replicates as detailed in the figure legends.
Randomization	No randomization was performed, since this study did not allocate experimental groups.
Blinding	No blinding was performed, since it is not relevant to this study.

Reporting for specific materials, systems and methods

We require information from authors about some types of materials, experimental systems and methods used in many studies. Here, indicate whether each material, system or method listed is relevant to your study. If you are not sure if a list item applies to your research, read the appropriate section before selecting a response.

Materials & experimental systems

n/a	Involved in the study
☐	☒ Antibodies
☒	☐ Eukaryotic cell lines
☒	☐ Palaeontology and archaeology
☒	☐ Animals and other organisms
☒	☐ Clinical data
☒	☐ Dual use research of concern

Methods

n/a	Involved in the study
☒	☐ ChIP-seq
☒	☐ Flow cytometry
☒	☐ MRI-based neuroimaging

Antibodies

Antibodies used

Anti-FKBP52 (monoclonal), Hi52b, 1:1000 dilution, a gift from Dr. Marc Cox, The University of Texas at El Paso
Anti-PGK1 (monoclonal, Invitrogen #459250), Clone 22C5D8, RRID AB_2532235, 1:10000 dilution

Validation

For Anti-FKBP52, see: Riggs, D. L. et al. The Hsp90-binding peptidylprolyl isomerase FKBP52 potentiates glucocorticoid signaling in vivo. The EMBO Journal 22, 1158-1167, doi:10.1093/emboj/cdg108 (2003).
Anti-PGK1 has been validated by Western blot using a range of different dilutions in ~200 publications, see: https://www.thermofisher.com/antibody/product/PGK1-Antibody-clone-22C5D8-Monoclonal/459250
