# Supplementary figures and images for: Cryo-EM reveals how Hsp90 and FKBP immunophilins co-regulate the glucocorticoid receptor
Source: Nat Struct Mol Biol. 2023 Nov 9;30(12):1867–77. doi: 10.1038/s41594-023-01128-y (PMC10716051; doi:10.1038/s41594-023-01128-y)

Source Data of Full-length, Uncropped Gels and Blots  
Extended Data Fig. 1

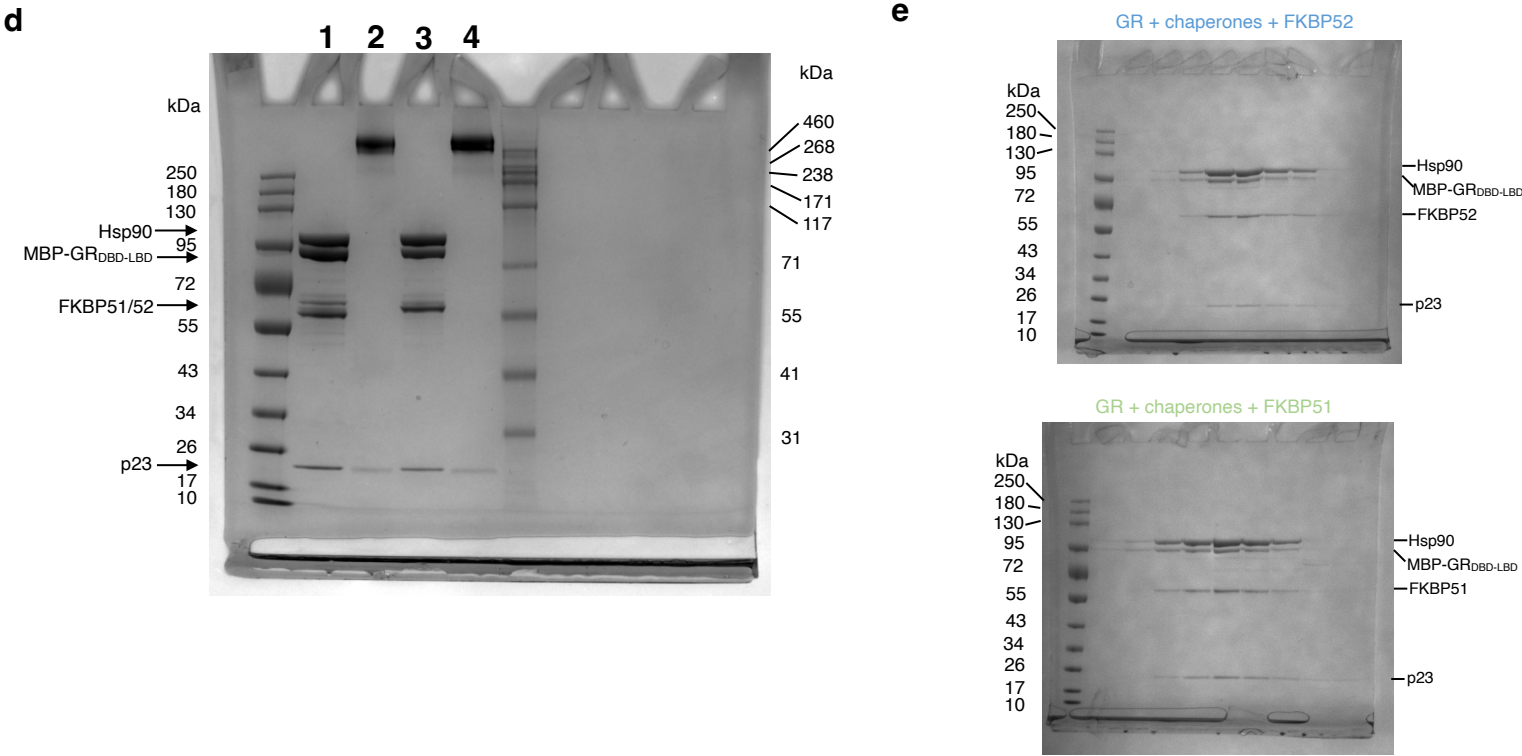

Extended Data Fig. 5

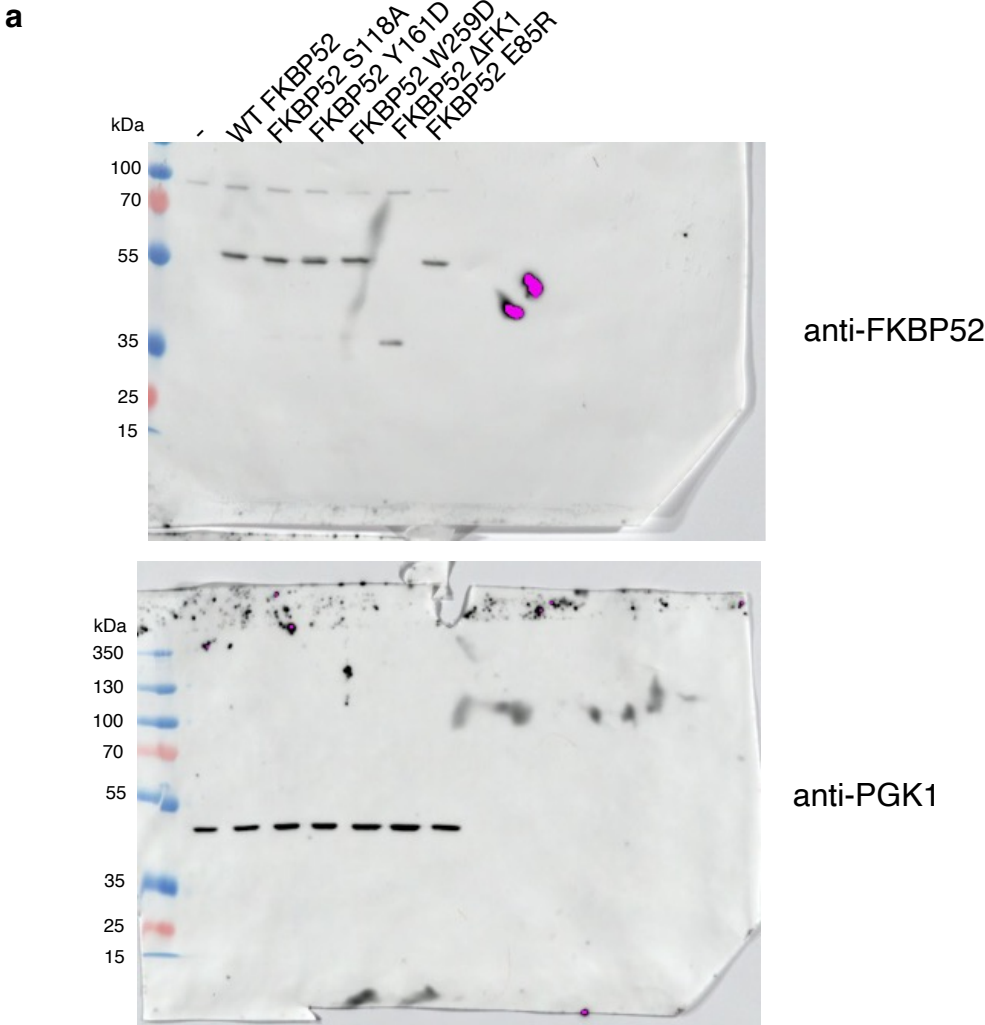

Supplement: Supplementary file 10 — Unprocessed western blots and gels, labeled for each extended data figure. [file 41594_2023_1128_MOESM10_ESM.pdf]
